# Castleman disease mimicked pancreatic carcinoma: report of two cases

**DOI:** 10.1186/1477-7819-10-154

**Published:** 2012-07-23

**Authors:** Hua Guo, Yan Shen, Wei-Lin Wang, Min Zhang, Hong Li, Ying-Sheng Wu, Sheng Yan, Xiao Xu, Jian Wu, Shu-Sen Zheng

**Affiliations:** 1 Division of Hepatobiliary and Pancreatic Surgery, Department of Surgery First Affliliated Hospital,School of Medicine, Zhejiang University, 79 Qingchun Street, Zhejiang Province, Hangzhou, 310003, China; 2 Key laboratory of Combined Multi-organ Transplantation, Ministry of Public Health, 79 Qingchun Street, Zhejiang Province, Hangzhou, 310003, China; 3 Key laboratory of Organ Transplantation of Zhejiang Province, 79 Qingchun Street, Zhejiang Province, Hangzhou, 310003, China

**Keywords:** Castleman disease, Peripancreatic tumor, Hyaline-vascular type

## Abstract

Castleman disease (CD) is an uncommon benign lymphoproliferative disorder, which usually presents as solitary or multiple masses in the mediastinum. Peripancreatic CD was rarely reported. Herein, we report two cases of unicentric peripancreatic CD from our center. A 43-year-old man and a 58-year-old woman were detected to have a pancreatic mass in the routine medical examinations. Both of them were asymptomatic. The computed tomography and ultrasonographic examination revealed a mild enhancing solitary mass at the pancreatic head/neck. No definite preoperative diagnosis was established and Whipple operations were originally planned. The intraoperative frozen section diagnosis of both patients revealed lymphoproliferation. Then the local excisions of mass were performed. Histological examination revealed features of CD of hyaline-vascular type. No recurrence was found during the follow-up period. CD should be included in the differential diagnosis of pancreatic tumors. Local excision is a suitable surgical choice.

## Background

Castleman disease (CD) is an uncommon benign lymphoproliferative disorder of unknown etiology. It was first described by Castleman in 1956 [1]. There are three pathologic variants: hyaline vascular CD, plasma cell CD, and mixed type of CD is characterized that a patient had features of both hyaline vascular and plasma cell types of CD [2]. Plasmablastic variant of CD, which was considered as a subvariant of plasma cell type, occurs predominantly in immunosuppressed patients and human immunodeficiency virus (HIV)-positive patients [3]. Clinically, CD may present in the forms of unicentric and multicentric. The unicentric variant of CD (UCD) is the most common form of the disease, which is confined to a single lymph-node chain or area, with hyaline vascular type. It is often asymptomatic and curable by surgical excision of the mass. The multicentric variant of CD (MCD) is a less common but more aggressive form. Its corresponding histological pattern is usually the plasma cell and mixed type [3]. Unicentric peripancreatic CD was rarely reported in the published literature. Herein, we report two cases of unicentric peripancreatic CD of hyaline vascular type from our center.

## Case presentation

**Figure 1:**
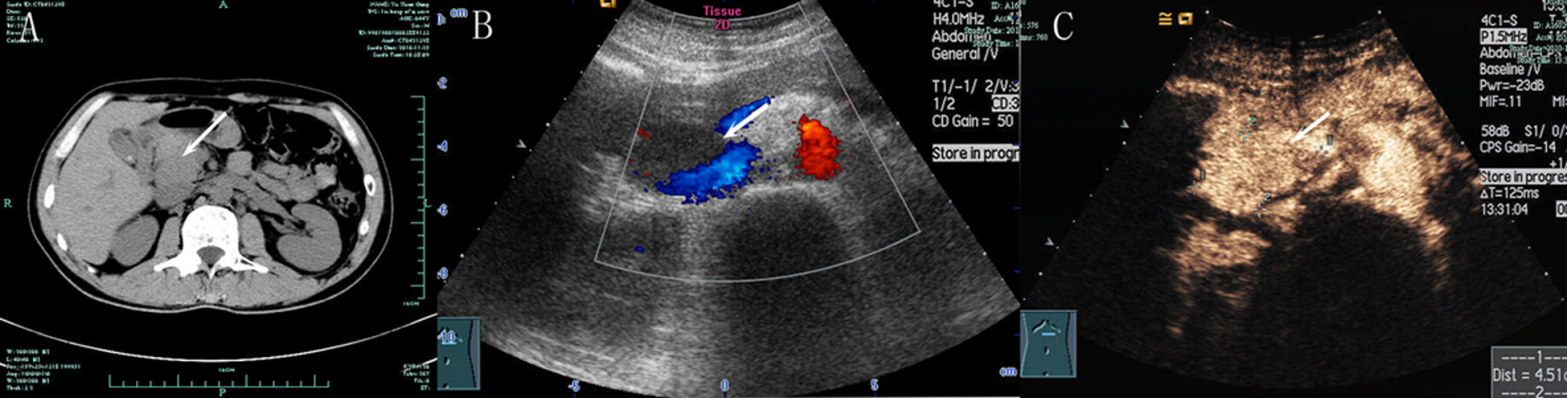
**Image findings of case 1.** Case1: Axial unenhanced computed tomography of abdomen depicts a 3 × 2.1cm mass (arrows) at pancreatic head (**A**). Contrast-enhanced ultrasonography reveals a solitary mass that homogeneous intense enhancement after contrast material administration (**B,C**).

Case 1: a 43-year-old man visited to us with an abdominal mass detected by ultrasonographic examination in a routine medical examination. He had a history of IgA nephropathy for 1 year. The preoperative serum creatinine was slightly elevated. The patient was asymptomatic with a normal appetite, no vomiting, no abdominal pain, no jaundice, and no weight loss. The tumor markers CEA, AFP, and CA125 were normal, the CA 19–9 was 54.8 U/mL(normal range, 0–37 U/mL). Chest X-ray was normal. As the serum creatinine was slightly elevated, the unenhanced computed tomography (CT) and contrast-enhanced ultrasonography were performed, the result showed a 3 × 2.1 cm, well-demarcated, mass at the pancreatic head (Figure 1).

**Figure 2:**
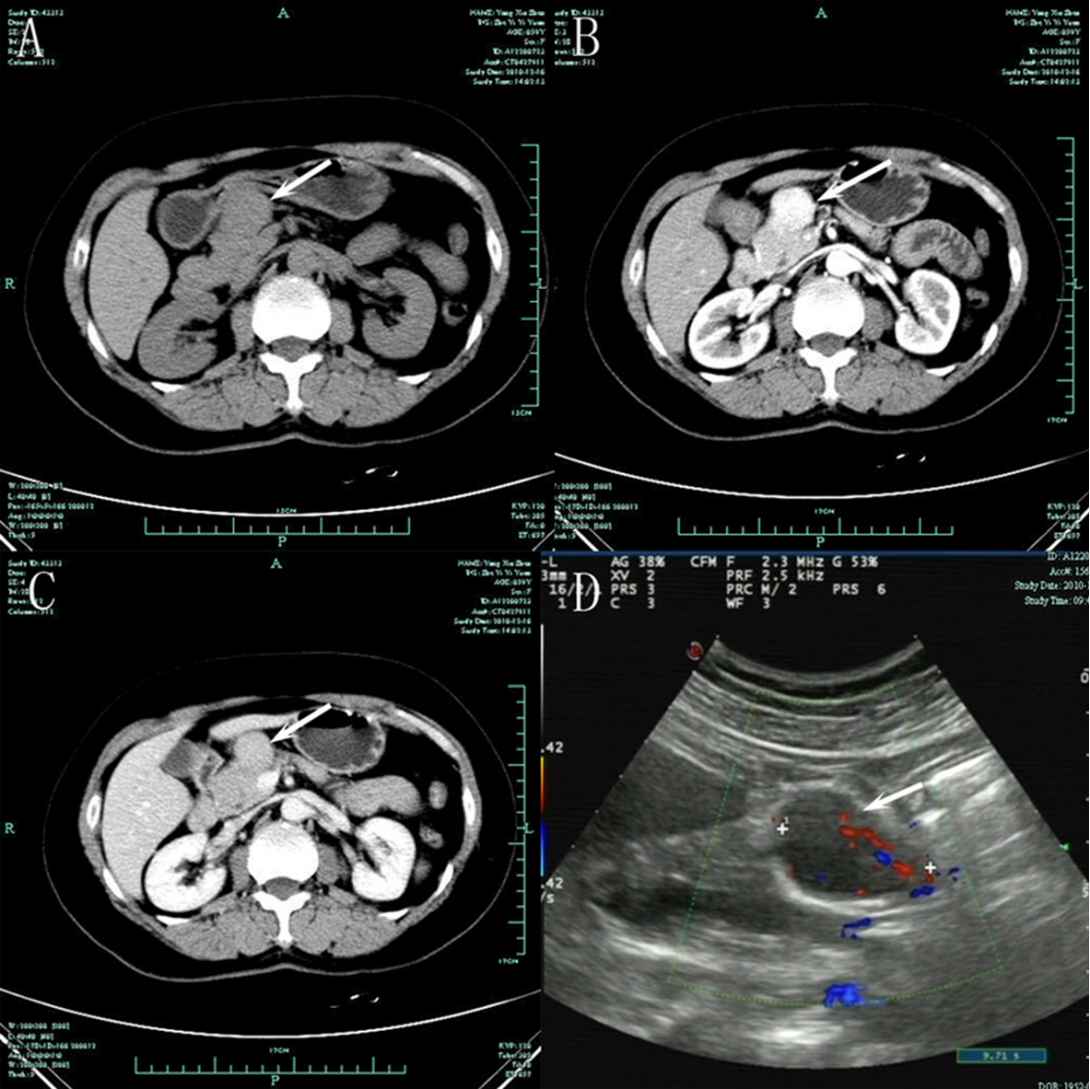
**Image findings of case 2.** Case 2: Axial contrast-enhanced CT image of the abdomen depicts a 4 × 2.7 cm moderately enhancing mass (arrows) at pancreatic neck. Axial unenhanced (**A**), arterial phase (**B**), and delayed phase (**C**) CT images of the abdomen and ultrasonography (**D**).

Case 2: a 58-year-old woman with no remarkable medical history visited our hospital with a mass detected by ultrasonographic examination in a routine examination. This patient was also asymptomatic. Laboratory data were within normal limits and a chest X-ray was normal. Tumor markers CEA, AFP, and CA125 were normal, the CA 19–9 was 46.4 U/mL. Contrast-enhanced CT showed a 4 × 2.7 cm, well-demarcated, mild enhancing mass at the pancreatic neck (Figure 2).

**Figure 3:**
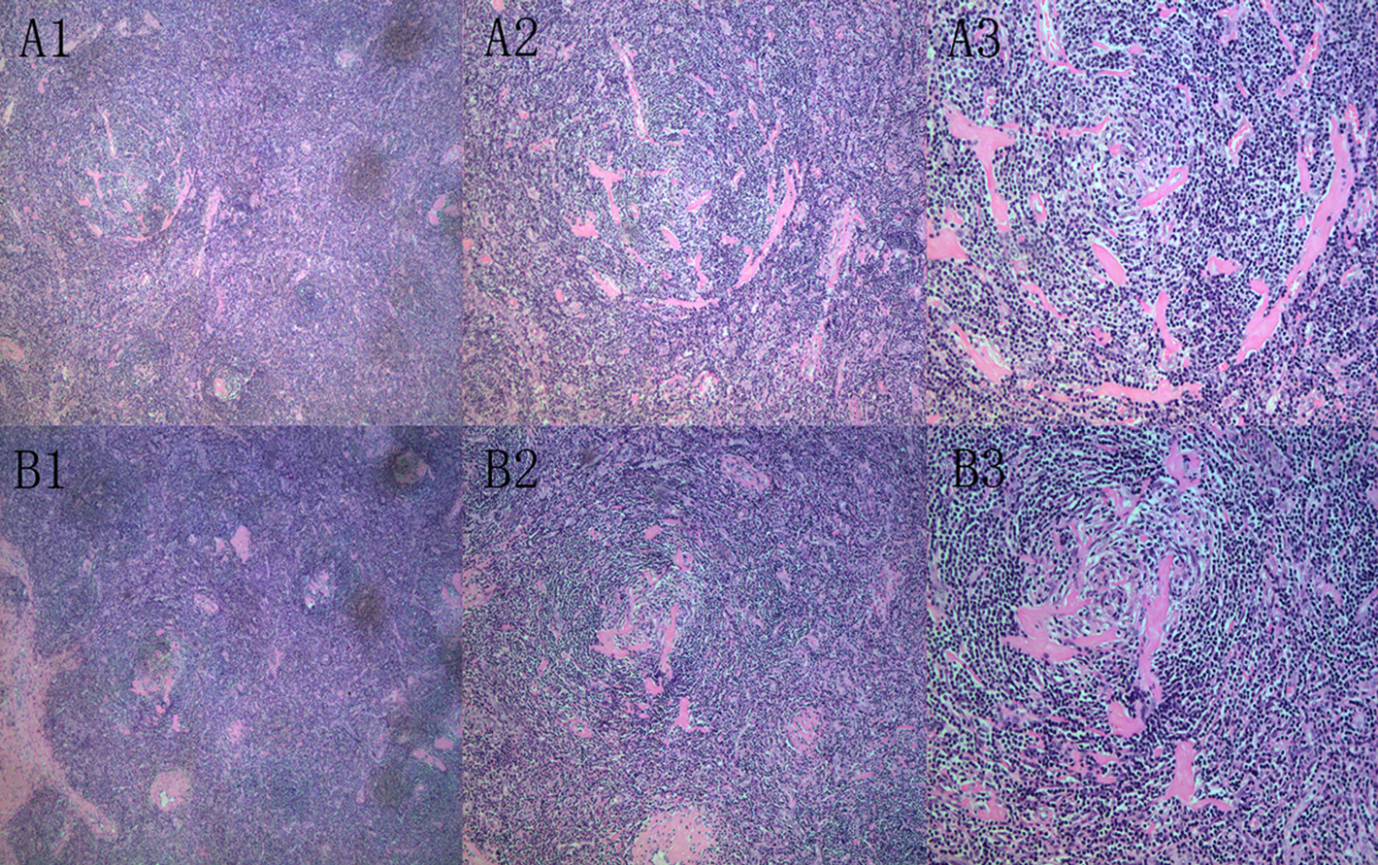
**Histological findings.** Typical histologic features of Castleman disease with hyaline vascular variant(case1:A1-A3; case2 B1-B3) hematoxylin-eosin stained photo-micrograph shows typical paracortical expansion with mixed inflammatory cells, including mature lymphocytes and plasma cells, and a prominent proliferation of blood vessels(original magnification A1,B1: ×40; A2,B2: ×100; A3,B3: ×200). High-power photomicrograph (original magnification,×200) of one area shows a germinal center with the classic ‘onionskin’ appearance.

Both patients were asymptomatic. The image findings revealed a mass in pancreas, preoperative tumor marker CA 19–9 were slightly elevated. Both were mimicked carcinoma of pancreas, and Whipple operations were planned before the operation. Intraoperatively, the masses were found closely adhere to pancreas. The masses were encapsulated and well demarcated from the attached pancreatic tissue. Thorough exploration revealed no intra-abdominal lymphadenopathy or any visceral abnormalities. The intraoperative frozen section diagnosis of both patients revealed lymphoproliferation. Then the original plan was changed, the local excisions of the masses were performed to avoid a more morbidity manner of the Whipple operation. The postoperative histological examination revealed typical features of the hyaline-vascular type of CD. Hematoxylin-eosin (HE) stains showed typical paracortical expansion with mixed inflammatory cells, and a prominent proliferation of blood vessels. High-power photomicrograph of one area showed a germinal center with the classic ‘onionskin’ appearance (Figure 3). The tumor markers were re-examined 1month after the operation, and the CA 19–9 in both patients were within normal range.

## Discussion

The etiology and pathophysiologic basis of CD remains unclear until now. The chronic inflammation, immunodeficiency state, and autoimmune disorders (such as viral infection involving the human herpes virus 8, the Epstein-Barr virus, or autoimmune hemolytic anemia) are considered to be the possible causal factors of CD. The dysregulation of inflammatory mediators, particularly interleukin-6 (IL-6) secretion has become the leading theory explaining constitutional symptoms and laboratory abnormalities [4,5]. Of the two patients, case1 was diagnosed as IgA nephropathy which considered an autoimmune disease, and received steroid treatment, but case2 had no inflammatory or autoimmunity disorder. Komatsuda *et al*. [6] reported a patient who developed UCD during the course of immunosuppressive therapy for IgA nephropathy associated with cutaneous nodules, the symptoms and abnormal laboratory findings were improved after anti-interleukin-6 receptor antibody administration. Whether case1 with peripancratic CD was associated with IgA nephropathy or immunosuppressive therapy was uncertain.

Clinically, CD is usually divided into unicentric and multicentric types. The UCD is most common with Hyaline vascular type. The mass is usually asymptomatic and localized, and is discovered incidentally, although some patients present with fever, fatigue, and localized pressure symptoms from the mass. The histological findings are of a multiple germinal center surrounded by circumferentially arranged layers in an onionskin pattern, of small lymphocytes, with a prominent vascular stroma and occasional plasma cells, which are characteristic of hyaline-vascular disease.

The preoperative diagnosis of the CD is very difficult. CD most commonly presents as a solitary mass. The classic CT appearance of hyaline vascular CD is that of a solitary enlarged lymph node or localized nodal mass that demonstrate homogeneous intense enhancement after contrast material administration. Diagnostic image sometimes mimics malignancy and CD cannot be identified because of the lack of disease specific signs. Some case reports had introduced an endoscopic ultrasound-guided fine needle aspiration (EUS-FNA) to establish the diagnosis of CD preoperatively [7,8], however, the sensitivity and specificity of this manner still had not been confirmed by large sample random clinical trial. The diagnosis of CD was still mostly dependent on the postoperative histological examination. Our two patients were both mimicked as carcinoma of pancreas so the preoperative cytologic examinations were not performed.

Surgery, radiotherapy, steroids, immunotherapy (interferon-α or anti-IL-6 antibodies), and combination chemotherapy have all been used to manage the disease [4]. Complete surgical excision remains the most used treatment strategy for unicentric CD, which confers a cure rate approaching 100% [9]. When the lesion is completely removed the prognosis is always perfect. If complete resection is difficult, partial resection may also be helpful, the recurrence rate after subtotal resection is also low [10]. Recurrence of CD after surgical excisions is rarely reported, even if the patients show recurrence, the disease remains progression-free after a repeat resection [11]. Extended surgery, which might cause more morbidity, is not required because CD was considered as a benign procedure. Radiotherapy or chemotherapy may be helpful, but it is not curative and the results vary [12]. Neoadjuvant use of rituximab [13] and neoadjuvant radiotherapy [14] have been reported in the treatment of irresectable unicentric CD to reduce tumor size and vascularity to allow for a less morbid surgical resection. In the two patients, during the intraoperative exploration, the occupation was found solitary, and the masses were encapsulated and well demarcated from the attached pancreatic tissue. The intraoperative frozen section diagnosis revealed lymphoproliferation. So the original surgical plans were changed, the Whipple resectiongiven up, and the mildmorbidity local excisions were performed,respectively. When the pathology results were available, chest CT scan and neck ultrasound scan were tested to confirm the tumors were unicentric. The postoperative courses were uneventful. No recurrence was found during the follow-up period. Interestingly, preoperative CA 19–9 of the two patients were slightly elevated, and the CA 19–9 levels were reduced to normal range after the tumor excision. Whether the elevation of serum CA 19–9 level correlated with the CD needs more evidences.

## Conclusions

In conclusion, CD should be included in the differential diagnosis of pancreatic tumors. Preoperative and intraoperative differential diagnosis of CD is important. The less morbidity local excision is a suitable surgical choice.

## Consent

Written informed consent was obtained from the patients for publication of this report and any accompanying images. A copy of the written consent is available for review by the Editor-in-Chief of this journal.

## Competing interest

The authors declare that they have no competing interests.

## Authors’ contributions

Guo H had the idea of case report and wrote the main manuscript. Wang WL, Shen Y, and Zhang M performed the operations. Wu YS, Yan S, Xu X, and Wu J collected the patients’ data and follow-up radiological images. Li H participated in the manuscript preparation. Zheng SS revised the manuscript for important intellectual content, and gave the final approval for the version to be submitted for publication. All authors read and approve the final manuscript.
